# Editorial: Developing personalized treatment in neurodegenerative disorders: role of genomics and novel technologies in identifying actionable targets and developing interventions in rare-diseases

**DOI:** 10.3389/fnins.2024.1403015

**Published:** 2024-04-09

**Authors:** Anja Kovanda, Sabina Vatovec, Valentino Rački

**Affiliations:** ^1^Clinical Institute of Genomic Medicine, University Medical Centre Ljubljana, Ljubljana, Slovenia; ^2^Faculty of Medicine, University of Ljubljana, Ljubljana, Slovenia; ^3^Novartis, Ljubljana, Slovenia; ^4^Faculty of Medicine, University of Rijeka, Rijeka, Croatia

**Keywords:** personalized treatment, genomics, actionable targets, neurodegenerative diseases, whole genome sequencing (WGS), exome sequencing (ES), glycomics mass spectrometry, deep brain stimulation (DBS)

Neurodegenerative diseases represent one of the leading causes of disability and death in the developed world and include rare diseases and common diseases with unresolved causality, such as Alzheimer's and Parkinson's disease.

Traditionally, the development of novel therapies involves a decade-long and prohibitively costly process, especially challenging because it attempts to simultaneously target genetically heterogeneous diseases. We aimed to identify how novel genomic technologies can be used both to better identify causes of neurological disease and those patients most likely to benefit from interventions, which are the first steps toward developing personalized treatment.

The works by Lee et al. and Peng et al. highlight how new technologies, or technologies used in new ways, may identify novel causes of disease, biomarkers, and information on disease pathology. Lee et al. have attempted to identify additional genetic causes of schizophrenia, which is a high-burden heterogeneous psychiatric disorder with missing heritability, by using third-generation genome sequencing to test families with severe chronic schizophrenia. Using whole genome sequencing and bioinformatics tools to compare the probands and healthy controls, Lee et al. identified medium-sized structural variants that are not detectable by other technologies in genes involved in brain development, neuronal/synaptic function, learning/memory, and hearing. Such research provides a novel hypothesis on the heritability of this complex and debilitating neurological disease, as well as provides a rationale for re-testing existing disease cohorts using novel technologies, such as whole genome sequencing.

The review by Peng et al. provides an overview of the methods and applications currently in use for glycomics. Glycomics have the potential to provide novel biomarkers and information on disease pathology in neurological disease, as glycosylation represents a vital post-translational modification mediating many biological functions. Peng et al. showcase the different ways in which mass spectrometry may be used to identify and quantify glycans involved in neurological diseases and list the known markers already identified in clinical nervous system diseases, focusing our attention on how non-genetic methods may be used for the assessment of neurological patients or effectiveness of therapy, from blood or CNS.

The perspectives by Ou et al. and Rački et al. focus on the personalization of treatment in neurodegenerative disorders. Ou et al. show, by using exome sequencing to identify carriers of pathogenic variants in the GBA1 gene in Parkinson's disease, that individual genes may be linked with a predisposition to certain symptoms, in this case, freezing of gait. Freezing of gait is independently associated with worse overall scores, so identifying associated genetic variants is important for the timely quality of life interventions, counseling, and participation in novel treatment strategies. The perspective by Rački et al. aims to show how genetic testing may be useful to identify patients most likely to benefit from deep brain stimulation. Deep brain stimulation is a neuromodulatory technique used for Parkinsonism and dystonia symptoms. Rački et al. review the findings on the association of which variants and genes have so far been associated with a favorable outcome of deep brain stimulation. The perspective also presents two patient cases, that show, despite all of the remaining challenges, genetics today has the potential to refine patient selection and enhance treatment outcomes in neurodegenerative disorders.

As can be understood from the publications presented here, the field of neurology is entering the era of personalization in the identification of novel causes of disease, as well as in the characterization and assessment of disease progression using genetic and other methods. Furthermore, novel technologies, be it genetic, such as exome and genome sequencing, that are explored in detail in other recent Research Topic (Mao et al., 2023), or other, such as mass spectroscopy, are available and accessible and can be used to guide us in our decision to treat, as well as to follow-up on the effectiveness of the intervention. While for the moment, it appears that personalization is still mostly performed in the more common neurodegenerative disorders, such as Parkinson's disorder, its potential to identify patients most likely to benefit and thus enhance treatment outcomes will hopefully advance such efforts in other rarer neurological diseases in the near future.

## Author contributions

AK: Writing – original draft, Writing – review & editing. SV: Writing – original draft, Writing – review & editing. VR: Writing – original draft, Writing – review & editing.
